# The genomic response to 20-hydroxyecdysone at the onset of *Drosophila *metamorphosis

**DOI:** 10.1186/gb-2005-6-12-r99

**Published:** 2005-11-21

**Authors:** Robert B Beckstead, Geanette Lam, Carl S Thummel

**Affiliations:** 1 Department of Human Genetics, Howard Hughes Medical Institute, University of Utah School of Medicine, Salt Lake City, UT 84112-5331, USA

## Abstract

**Background:**

The steroid hormone 20-hydroxyecdysone (20E) triggers the major developmental transitions in *Drosophila*, including molting and metamorphosis, and provides a model system for defining the developmental and molecular mechanisms of steroid signaling. 20E acts via a heterodimer of two nuclear receptors, the ecdysone receptor (EcR) and Ultraspiracle, to directly regulate target gene transcription.

**Results:**

Here we identify the genomic transcriptional response to 20E as well as those genes that are dependent on *EcR *for their proper regulation. We show that genes regulated by 20E, and dependent on *EcR*, account for many transcripts that are significantly up- or downregulated at puparium formation. We provide evidence that 20E and EcR participate in the regulation of genes involved in metabolism, stress, and immunity at the onset of metamorphosis. We also present an initial characterization of a 20E primary-response regulatory gene identified in this study, *brain tumor *(*brat*), showing that *brat *mutations lead to defects during metamorphosis and changes in the expression of key 20E-regulated genes.

**Conclusion:**

This study provides a genome-wide basis for understanding how 20E and its receptor control metamorphosis, as well as a foundation for functional genomic analysis of key regulatory genes in the 20E signaling pathway during insect development.

## Abstract 2

The genome-wide transcriptional response to 20-hydroxyecdisone at the onset of *Drosophila *metamorphosis, as well as its dependency on one of the ecdysone receptors is described.

## Background

Small lipophilic hormones such as retinoic acid, thyroid hormone, and steroids control a wide range of biological pathways in higher organisms. These hormonal signals are transduced into changes in gene expression by members of the nuclear receptor superfamily that act as hormone-responsive transcription factors [1]. Although extensive studies have defined the molecular mechanisms by which nuclear receptors regulate transcription, much remains to be learned about how these changes in gene activity result in the appropriate biological responses during development.

*Drosophila melanogaster *provides a powerful model system for elucidating the molecular and genetic mechanisms of hormone action. Pulses of the steroid hormone 20-hydroxyecdysone (20E) act as critical temporal signals that direct each of the major developmental transitions in the *Drosophila *life cycle, including molting and metamorphosis [2]. A high titer pulse of 20E at the end of the third larval instar triggers puparium formation, initiating metamorphosis and the prepupal stage of development. A second 20E pulse approximately 10 hours after pupariation triggers adult head eversion and marks the prepupal-to-pupal transition. Our current understanding of the molecular mechanisms of 20E action in insects derives from detailed characterization of the puffing patterns of the giant larval salivary gland polytene chromosomes [3-6]. These studies exploited an organ culture system that allows the use of defined hormone concentrations as well as the addition of cycloheximide to distinguish primary responses to the 20E signal [5,6]. The puffing studies revealed that 20E acts, at least in part, through a two-step regulatory cascade. The hormone directly induces approximately six early puff genes [7]. The protein products of these genes were proposed to repress their own expression as well as induce many secondary-response late puff genes that, in turn, were assumed to direct the appropriate biological responses to the hormone.

The identification and characterization of a 20E receptor, along with several early and late puff genes, has supported and extended this hierarchical model of 20E action. Like vertebrate hormones, 20E regulates gene expression by binding to a nuclear receptor heterodimer, consisting of the ecdysone receptor (EcR) and Ultraspiracle (USP), which are orthologs of the vertebrate LXR and RXR receptors, respectively [8]. Several early puff genes have been identified, including the *Broad-Complex *(*BR-C*) and *E74 *[9,10]. As predicted by the puffing studies, these genes encode transcription factors that directly regulate late puff gene expression [11,12] and are essential for appropriate biological responses to 20E [13,14]. Other studies, however, have shown that not all early puffs encode transcriptional regulators. These include a calcium binding protein encoded by *E63-1 *[15] and the *E23 *ABC transporter gene [16]. In addition, a molecular screen identified fifteen new 20E primary-response genes, only two of which correspond to early puff loci, suggesting that the hormone triggers a much broader transcriptional response than is evident from the puffing pattern of the salivary gland polytene chromosomes [17]. Similarly, the isolation of late puff genes has demonstrated that some of these presumed effectors may function in a regulatory capacity, such as the CDK-like protein encoded by *L63 *[18].

Several papers have used microarrays to identify genes that change their expression at the onset of metamorphosis [19-21]. Although critical for understanding the dramatic switches in gene expression that occur at this stage, these studies are restricted to developmental analysis of staged tissues or animals, with no direct links to 20E signaling. Increasing evidence indicates that other hormones and receptors may contribute to the complex developmental pathways associated with metamorphosis [8,22,23]. In addition, some transcripts are induced at puparium formation independently of 20E or its receptor [24]. It thus remains unclear to what extent 20E and EcR contribute to the global reprogramming of gene activity that occurs at the early stages of metamorphosis.

In this study, we use larval organ culture in combination with microarray technology to identify genes regulated by 20E alone or 20E in the presence of cycloheximide [5,6]. We also examine the effects of disrupting *EcR *function on the global patterns of gene expression at the onset of metamorphosis, and use these data to refine our lists of 20E-regulated genes. The top 20E-regulated genes described here include many of the key genes identified by puffing studies, validating our approach. We also identify many new genes that are part of the 20E/EcR regulatory cascade and define roles for EcR in the regulation of stress, immunity, and metabolism at the onset of metamorphosis. Finally, we characterize one 20E primary-response target in more detail - the *brat *gene, which encodes a translational regulator [25,26]. We show that *brat *mutants display defects at the onset of metamorphosis and mis-regulate key 20E target genes, consistent with a disruption in 20E signaling. This work provides a genomic foundation for defining the roles of 20E and EcR in controlling insect development.

## Results and discussion

### Most genes that change expression at pupariation require *EcR *function

**Figure 1:**
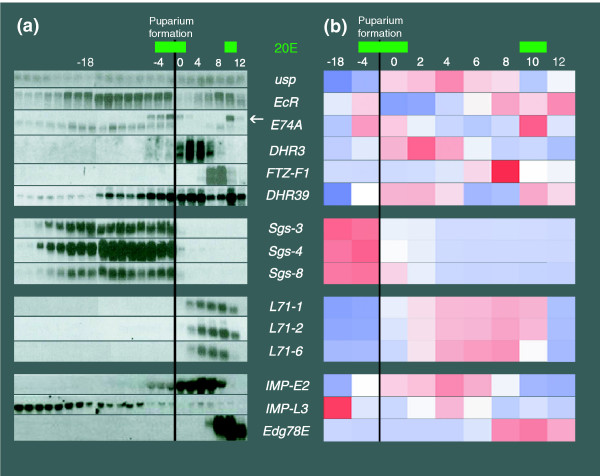
Validation of the temporal patterns of 20E-regulated gene expression as determined by microarray analysis. **(a) **Northern blot hybridizations adapted with permission from published data [27,28]. Arrow indicates *E74A *isoform. **(b) **Cluster analysis of microarray data derived from RNA samples isolated from staged wild-type animals. The colors for each time point represent the change in the level of expression relative to the average expression levels across all time points for that gene, with dark blue indicating the lowest level of expression and red indicating the highest level, as depicted on the bottom. The numbers at the top indicate hours relative to pupariation, with green bars representing the peaks of 20E titer.

To identify genes that alter their expression in synchrony with the late third instar and prepupal pulses of 20E, RNA was isolated from *w*^*1118 *^animals staged at -18, -4, 0, 2, 4, 6, 8, 10, and 12 hours relative to pupariation, labeled, and hybridized to Affymetrix *Drosophila *Genome Arrays. The sensitivity and accuracy of the array data were determined by comparing the expression patterns of known 20E-regulated genes with previously published developmental Northern blot data [27,28]. A subset of this analysis, depicted in Figure 1, reveals that the temporal expression pattern of key regulatory genes - *EcR*, *usp*, *E74A*, *DHR3*, *FTZ-F1*, and *DHR39 *- are faithfully reproduced in the temporal arrays, as well as the 20E-regulated switch from *Sgs *glue genes to *L71 *late genes in the larval salivary glands, and the expression of representative *IMP *and *Edg *genes in the imaginal discs and epidermis. This comparison demonstrates that the microarrays accurately reflect the temporal patterns of 20E-regulated gene expression at the onset of metamorphosis and have sufficient sensitivity to detect rare transcripts such as *EcR *and *E74A*.

**Figure 2:**
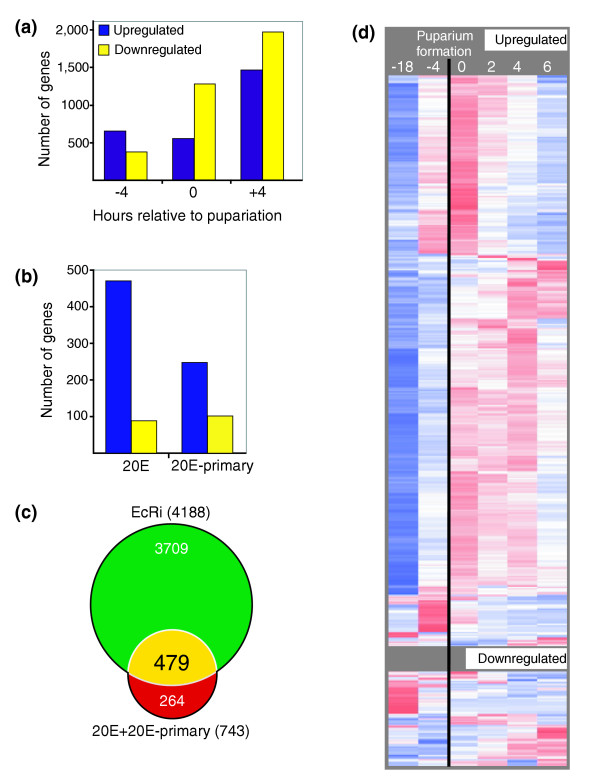
Microarray results for *EcRi *and 20E organ cultures experiments. **(a) **Graphic depiction of the number of genes upregulated (blue) or downregulated (yellow) in *EcRi *late third instar larvae or prepupae at the times indicated. **(b) **Graphic depiction of the number of 20E-regulated genes or 20E primary-response genes that are either upregulated (blue) or downregulated (yellow) in third instar larval organ culture. **(c) **Venn diagram depicting the overlap between all *EcR*-regulated genes and the combined 20E-regulated genes and 20E primary-response genes. **(d) **Cluster diagram depicting the temporal expression pattern of the 479 genes in the 20E-final set, divided into those genes that are upregulated by 20E (above) or downregulated by 20E (below). Times are shown in hours relative to puparium formation, and colors are as described in Figure 1b.

*EcR *mutants die during early stages of development, complicating their use for studying receptor function during metamorphosis. To circumvent this problem, we employed a transgenic system that allows heat-induced expression of double-stranded RNA corresponding to the *EcR *common region to disrupt *EcR *function at puparium formation (*EcRi*) [29]. RNA was harvested for array analysis from *EcRi *animals staged at -4, 0, and 4 hours relative to pupariation. All *EcRi *animals formed arrested elongated prepupae, consistent with an effective block in 20E signaling and highly reduced EcR protein levels (Figures 2 and 3c in [29]). Data obtained from these arrays were compared to our array data from control animals at the same stages of development to identify *EcR*-dependent genes. The initial effect of *EcR *RNA interference RNA (RNAi) is significant upregulation of gene expression in late third instar larvae, followed by a switch at puparium formation such that the majority of genes are not properly induced (Figure 2a). These data are consistent with genetic studies of *usp *that define a critical role for this receptor in repressing ecdysone-regulated genes during larval stages [30], and provide further evidence that one essential function for the EcR-USP heterodimer is to prevent premature maturation through the repression of select 20E target genes during larval stages.

A total of 4,188 genes change their expression at least 1.5-fold in at least one time point in *EcRi *animals (Additional data file 1), suggesting that almost a third of all genes require *EcR*, either directly or indirectly, for their proper regulation at the onset of metamorphosis. This number is consistent with the 2,268 genes that have been reported to change their expression at pupariation in one of five tissues examined: midgut, salivary gland, wing disc, epidermis, and central nervous system [20]. It is also similar to the 4,042 genes that change their expression at least 1.5-fold at pupariation in our temporal arrays. Of these 4,042 genes, 2,680 are affected in *EcRi *animals, supporting the proposal that EcR plays a major role in coordinating transcriptional responses at the onset of metamorphosis. Not all genes that change their expression at pupariation, however, are dependent on *EcR*. Several such transcripts were selected for validation by Northern blot hybridization (Additional data file 3). This is consistent with an earlier microarray study of *EcR*-regulated genes in the larval midgut [20]. This study found that of 955 genes that change their expression in wild-type midguts at the onset of metamorphosis, 672 genes are affected by an *EcR *mutation while 283 genes are unaffected, close to the proportion of *EcR*-independent genes identified by our study. This is also consistent with earlier studies that indicate that other signaling pathways are active at this stage in development. For example, the *miR-125 *and *let-7 *microRNAs are dramatically induced at puparium formation, in tight temporal synchrony with the 20E primary-response *E74A *mRNA, but do so in a manner that is independent of either 20E or *EcR *[24]. Similarly, α-ecdysone, the immediate upstream precursor of 20E, has critical biological functions [23,31,32], can activate the DHR38 nuclear receptor [22], and can induce genes in *Drosophila *third instar larvae that are distinct from those that respond to 20E (RBB, GL and CST, unpublished results). The sesquiterpenoid juvenile hormone can also function with 20E to direct specific transcriptional responses during early metamorphosis [33-35]. The results of the study described here, however, indicate that most genes that change their expression at the onset of metamorphosis do so in an *EcR*-dependent fashion, and pave the way for future studies that integrate these responses with those of other signaling pathways.

### Identification of 20E-regulated genes in cultured larval organs

To identify 20E-regulated genes, wandering third instar larvae were dissected and their organs cultured in the presence of either no hormone, 20E alone, cycloheximide alone, or 20E plus cycloheximide for 6 hours. RNA extracted from these samples was analyzed on Affymetrix *Drosophila *Genome Arrays. Comparison of the no hormone and 20E-treated datasets led to the identification of 20E-regulated genes, while comparison of the cycloheximide dataset with data derived from organs treated with 20E and cycloheximide led to the identification of a set of genes we refer to as 20E primary-response genes. In comparing these datasets, it is important to note that cycloheximide treatment alone can stabilize pre-existing mRNAs and thus mask their induction by 20E [6,17,36]. These transcripts would not be identified by our experiments. In addition, some 20E-inducible genes are expressed at higher levels in the absence of protein synthesis, due to the lack of 20E-induced repressors [6,17]. The addition of cycloheximide thus provides a means of detecting 20E-regulated transcripts that might otherwise be missed. In this study, 743 20E-regulated genes were identified (Figure 2b), with 555 genes responding to 20E alone, 345 genes responding to 20E in the presence of cycloheximide (Additional data file 2), and 159 genes overlapping between these two datasets.

Comparison of the 20E-regulated genes to those genes that require *EcR *for their proper regulation at the onset of metamorphosis led to a final list of 20E-regulated, *EcR*-dependent genes. Only those genes that are upregulated by 20E in culture and downregulated in at least one of the *EcRi *time points, or downregulated by 20E in culture and upregulated in at least one of the *EcRi *time points, were considered for further analysis, leading to the identification of 479 genes (20E-final; Figure 2c). As depicted in Figure 2d, the majority of 20E-final genes that are upregulated by 20E are induced in -4 hour late larvae and/or early prepupae, in apparent response to the late larval 20E pulse, while many genes downregulated by 20E are repressed at these times. The downregulated 20E-final genes that peak in 4 to 6 hour prepupae could be repressed by 20E and thus expressed during this interval of low 20E titer.

We compared our *EcR*-dependent genes and the 20E-final gene set to data from two microarray studies that examined 20E-regulated biological responses - either *EcR*-dependent genes expressed in the larval midgut at pupariation [20] (Table 1, rows A and B), or changes in gene expression that occur during 20E-induced larval salivary gland cell death [37] (Table 1, rows C and D). As expected, many genes that are normally downregulated in the midgut at pupariation are upregulated in our *EcRi *gene set (113 genes; Table 1, row B), and genes that are normally upregulated in the midgut at pupariation are downregulated in our *EcRi *gene set (120 genes; Table 1, row A). Similarly, we see significant overlaps between our 20E-final set and midgut genes that change their expression at pupariation (65 genes upregulated and 10 genes downregulated; Table 1, rows A and B). Statistically significant overlaps were also observed with genes that change their expression during salivary gland cell death, consistent with a critical role for 20E in directing this response [37] (Table 1, rows C and D). These correlations validate our datasets and support the conclusion that our results represent 20E responses in multiple tissues at the onset of metamorphosis.

Those genes that are upregulated by 20E twofold or higher and dependent on *EcR *are listed in Table 2. An examination of this list reveals several known key mediators of 20E signaling during development. These include three classic ecdysone-inducible puff genes, *E74A*, *E75*, and *E78 *[7], as well as *Kr-h1*, which encodes a family of zinc finger proteins required for metamorphosis [38], the *DHR3 *nuclear receptor gene [39], and *Cyp18a1 *[17]. Expanding our list by including all 20E-regulated genes (Additional data file 2), results in the identification of the *DHR39*, *DHR78*, and *FTZ-F1 *nuclear receptor genes, as well as the *L71 *(*Eip71E*) late genes, *IMP-E2*, *IMP-L3*, *Fbp-2*, *Sgs-1*, *urate oxidase*, and numerous genes identified in other studies as changing their expression at the onset of metamorphosis [20,21,40]. The identification of well-characterized 20E-regulated genes within our datasets suggests that the other genes in these lists are also likely to function in 20E signaling pathways, and thus provide a foundation to extend our understanding of 20E action in new directions.

### Immunity, stress-response, and starvation genes are regulated by 20E at pupariation

**Figure 3:**
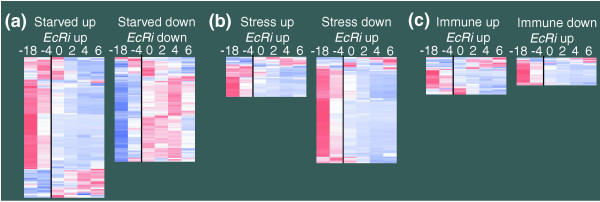
Temporal expression patterns of *EcR*-dependent genes that are regulated by **(a) **starvation, **(b) **stress, or **(c) **infection. Upregulated (up) and downregulated (down) genes are labeled, hours are relative to puparium formation, which is marked by a black line, and colors are as described in Figure 1b.

In an effort to identify biological pathways that might respond to 20E at the onset of metamorphosis, we compared our *EcRi *and 20E-final datasets with published microarray studies of circadian rhythm, starvation, stress, and immunity [41-45]. No statistically significant overlaps were seen with the circadian rhythm gene sets examined; however, we did observe significant overlaps with genes that are expressed during starvation, stress, or an innate immune response. For the starvation response, we examined genes that change their expression upon starvation for 4 hours or starvation in the presence of sugar for 4 hours [45]. We observed 120 genes induced under these conditions that are upregulated in *EcRi *animals, and 90 genes that are repressed upon starvation and downregulated in *EcRi *animals (Table 1, rows E and F). As shown in Figure 3a, the starvation-regulated genes are part of an *EcR*-dependent switch that occurs at puparium formation, where many of the induced genes are normally downregulated at puparium formation, and many starvation-repressed genes are upregulated at puparium formation. These genes include eight members of the cytochrome P450 family, three triacylglycerol lipase genes, α-trehalose-phosphate synthase, and a fatty-acid synthase gene that are downregulated at the onset of metamorphosis, while *lipid storage droplet-1*, *pumpless*, a UDP-galactose transporter, a lipid transporter, and phosphofructokinase are upregulated at this stage. Similarly, genes that change their expression in response to oxidative or endoplasmic reticulum stress [43] are significantly upregulated in *EcRi *animals at puparium formation (Table 1, rows G and H), reflecting their normal coordinate downregulation at puparium formation (Figure 3b), and demonstrating that this response is mediated by EcR. Within the 87 genes that overlap between the downregulated stress response genes and the upregulated *EcR*-dependent genes, we identified 14 of the 17 *Jonah *genes that encode a family of coordinately regulated midgut-specific putative proteases [46]. Six genes that encode trypsin family members are also within this gene set, indicating that many peptidase family members are regulated by EcR. Taken together with the data on *EcR*-regulated starvation genes, these results indicate that EcR plays a central role in controlling metabolic responses at pupariation, directing the change from a feeding growing larva to an immobile non-feeding pupa.

Genes that change their expression upon microbial infection [44,47] are also significantly upregulated in *EcRi *animals at puparium formation (Table 1, rows I and J), and coordinately downregulated at pupariation (Figure 3c). Interestingly, we identified both the Toll ligand-encoding gene *dorsal *and the key Toll effector gene *spätzle *as downregulated at the onset of metamorphosis in a *EcR*-dependent manner, suggesting that central regulators of the Toll-mediated immune response pathway are under EcR control [48]. In addition, well studied immune response genes are downregulated by 20E, including *Cecropin C*, *Attacin A*, *Drosocin*, *Drosomycin*, and *Defensin *(Additional data file 2). These observations indicate that many metabolic and immunity-regulated genes are part of the genetic program directed by 20E at the onset of metamorphosis, and that these genes are normally coordinately downregulated at puparium formation in an *EcR*-dependent manner.

### Identification of novel 20E primary-response regulatory genes

**Figure 4:**
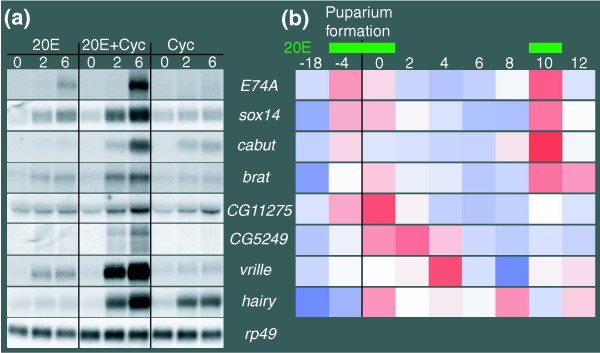
Validation of seven 20E primary-response regulatory genes. **(a) **Northern blot analysis of RNA samples isolated from organ cultures treated with either 20E alone, 20E plus cycloheximide (20E+Cyc), or cycloheximide (Cyc) alone, for 0, 2, or 6 hours. **(b) **Temporal expression patterns of depicted genes with hours shown relative to puparium formation. Green bars represent the peak 20E titers. Colors in the cluster analysis are as described in Figure 1b. Hybridization to detect *E74A *and *rp49 *mRNAs was included as a control.

We selected all potential transcriptional and translational regulators from the list of most highly induced 20E primary-response genes that are *EcR*-dependent (Table 2) and not yet implicated in 20E signaling pathways, identifying seven genes: *sox box protein 14 *(*sox14*), *cabut*, *CG11275*, *CG5249*, *vrille*, *hairy*, and *brain tumor *(*brat*). Northern blot hybridization was used to validate the transcriptional responses of these genes to 20E (Figure 4a). All seven genes are induced by 20E in larval organ culture, with *CG5249 *displaying a very low level of expression and *hairy *showing only a modest approximately twofold induction. Several transcripts are increased upon treatment with cycloheximide alone, consistent with its known role in stabilizing some mRNAs [6,17,36]. Addition of 20E and cycloheximide, however, resulted in higher levels of transcript accumulation, similar to the response seen when *E74A *is used as a control [6,49]. Their temporal patterns of expression at the onset of metamorphosis also reveal brief bursts of transcription that correlate with the 20E pulses that trigger puparium formation and adult head eversion (Figure 4b). These seven genes thus appear to represent a new set of 20E primary-response regulatory genes that could act to transduce the hormonal signal during metamorphosis.

### *brat *is required for genetic and biological responses to 20E during metamorphosis

**Figure 5:**
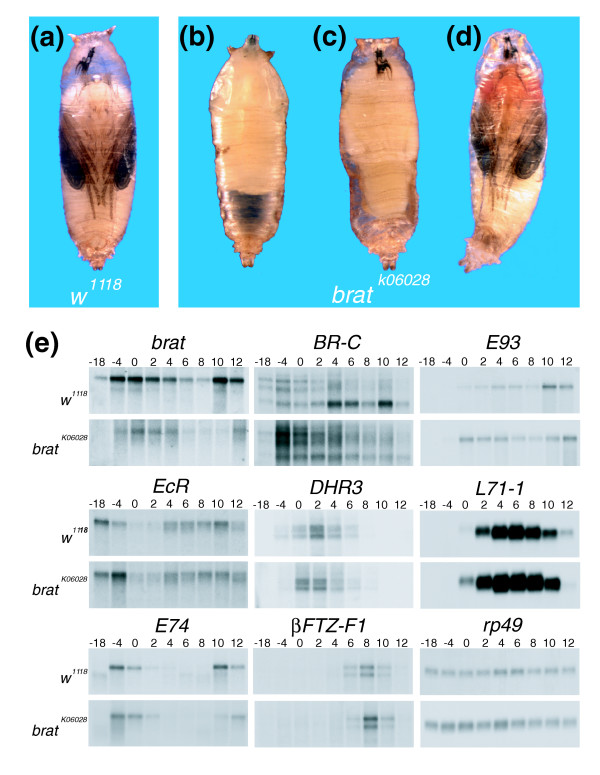
Mutations in *brat *lead to defects in genetic and biological responses to 20E. **(a) **Control *w*^*1118 *^pharate adult. **(b-d) **Representative *brat*^*k06028 *^mutant animals. **(e) **Northern blot analysis of *w*^*1118 *^and *brat*^*k06028 *^mutants staged in hours relative to pupariation. Blots were probed to detect *brat *mRNA and transcripts from seven different 20E-regulated genes. Hybridization to detect *rp49 *mRNA was included as a control for loading and transfer.

We examined roles for *brat *during metamorphosis because, unlike the other six 20E primary-response genes described above, a *brat *mutant allele is available (*brat*^*k06028*^) that allows an assessment of its functions during later stages of development [25,50]. The *brat*^*k06028 *^*P*-element maps to the fourth exon of the *brat *gene. Precise excisions of this transposon result in viable, fertile animals, demonstrating that the transposon is responsible for the mutant phenotype [25]. Lethal phase analysis of *brat*^*k06028 *^mutants revealed that 61% of the animals survive to pupariation, with the majority of these animals pupariating 1 to 2 days later than their heterozygous siblings (n = 400). Of those mutants that pupariated, 11% died as prepupae, 8% died as early pupae, 46% died as pharate adults, and the remainder died within a week of adult eclosion. Phenotypic characterization of *brat*^*k06028 *^mutant prepupae and pupae revealed defects in several ecdysone regulated developmental processes, including defects in anterior spiracle eversion (29%; Figure 5b–d), malformed pupal cases (15%, Figure 5b–d), and incomplete leg and wing elongation (12%). Northern blot hybridization of RNA isolated from staged *brat*^*k06028 *^mutant third instar larvae (Figure 5e, -18 and -4 hour time points) or prepupae (Figure 5e, 0 to 12 hours) revealed a disruption in the 20E-regulated transcriptional hierarchy. In wild type animals, *brat *mRNA is induced in late third instar larvae and 10 hour prepupae, similar to the temporal profile determined by microarray analysis (Figures 4b and 5e), with reduced levels of *brat *mRNA in *brat*^*k06028 *^mutants, consistent with it being a hypomorphic allele [25]. *βFTZ-F1 *is unaffected by the *brat *mutation in mid-prepupae, while *E74 *mRNA is reduced at 10 hours after pupariation (Figure 5e). *BR-C*, *E93*, *EcR*, *DHR3*, and *L71-1 *are expressed at higher levels in late third instar larvae and early prepupae (Figure 5e), with significant upregulation of *BR-C*. In addition, the smallest *BR-C *mRNA, encoding the Z1 isoform, is under-expressed in *brat *mutant prepupae (Figure 5e). It is unlikely that *brat *exerts direct effects on transcription since it encodes a translational regulator [26]. Nonetheless, these effects on 20E-regulated gene expression are consistent with the late lethality of *brat*^*k06028 *^mutants. In particular, the *rbp *function provided by the BR-C Z1 isoform is critical for developmental responses to 20E, and overexpression of BR-C isoforms can lead to lethality during metamorphosis [13,51]. Thus, not only are the *brat *mutant phenotypes consistent with it playing an essential role during metamorphosis, but it may exert this function through the regulation of key 20E-inducible genes. Efforts are currently underway to address the roles of the remaining six new 20E primary-response regulatory genes in transducing the hormonal signal at the onset of metamorphosis.

## Conclusions

The classic studies of the giant larval salivary gland polytene chromosomes established a new paradigm for the mechanisms of steroid hormone action, raising the exciting possibility that these hormones could act directly on the nucleus, triggering a complex regulatory cascade of gene expression [7,52]. Although subsequent molecular experiments confirmed and significantly expanded this hierarchical model of 20E action, no studies to date have addressed the genomic effects of 20E on gene regulation or the global effects of EcR on gene expression at the onset of metamorphosis. The work described here provides a new basis for our understanding of 20E signaling, returning to the genome-wide level of the original puffing studies, but identifying individual genes that act in this pathway. Much as earlier studies of puff genes provided a foundation for our understanding of steroid hormone action, we envision that future molecular and genetic characterization of 20E-regulated, *EcR*-dependent genes will expand our understanding of 20E action and insect maturation in new directions.

## Materials and methods

### Animals, staging, and phenotypic analysis

*w*^*1118 *^animals were used for phenotypic, array, and Northern blot studies. *brat*^*k06028*^/*CyO*, *kr-GFP *was used to analyze *brat *function. Third instar larvae were staged by the addition of 0.05% bromophenol blue to the food as previously described [53], or synchronizing animals at pupariation. *brat*^*k06028 *^animals were identified by the loss of the *kr-GFP *marker associated with the *CyO *balancer chromosome. For *EcR *RNAi, *hs-EcRi-11 *third instar larvae were heat-treated twice at 37°C, each time for 1 hour, at 24 hours and 18 hours prior to pupariation, as described [29]. RNA was harvested for microarray analysis at -4, 0, and 4 hours relative to pupariation from three independent collections of animals for each time point.

### Organ culture

Partial blue gut third instar larvae were staged by the addition of 0.05% bromophenol blue to the food, as previously described [53]. Eight animals were dissected in each well of a nine-well glass dish (Corning, Corning, NY, USA) and cultured in approximately 100 μl oxygenated Schneiders *Drosophila *Medium (Invitrogen, Carlsbad, CA, USA) at 25°C. Cultures were incubated in a styrofoam box under a constant flow of oxygen. Following an initial incubation of 1 hour, the medium was removed and replaced with either fresh Schneiders *Drosophila *Medium (no hormone), medium plus 8.5 × 10^-5 ^M cycloheximide (Sigma-Aldrich, St. Louis, MO, USA), medium plus 5 × 10^-6 ^M 20-hydroxyecdysone (Sigma), or medium plus cycloheximide and 20E, each for 6 hours at 25°C. Organs were collected and RNA was extracted as described below. All experiments were done in triplicate and harvested separately for microarray analysis.

### Microarray and cluster analysis

All experiments for microarray analysis were performed independently, in triplicate, to facilitate statistical analysis. Total RNA was isolated using TriPure (Roche, Indianapolis, IN, USA) followed by further purification with RNAeasy columns (Qiagen, Valencia, CA, USA). Probe labeling, hybridization to Affymetrix GeneChip^® ^*Drosophila *Genome Arrays (Affymetrix, Santa Clara, CA, USA), and scanning, were performed by the University of Maryland Biotechnology Institute Microarray Core Facility. dChip1.2 was used to normalize the raw data and determine gene expression values [54]. Statistically significant changes between sample sets were identified using significance analysis of microarray (SAM) with a delta value to give a <10% false discovery rate [55]. Further analysis and comparisons between datasets were performed using Access (Microsoft Corporation, Redmond, WA, USA). Cluster analysis was performed using dChip1.2. A cutoff of 1.3-fold change in expression level was used to restrict the 20E-regulated gene set (organ culture data), with a 1.5-fold cutoff for the *EcRi *gene sets. These fold cutoffs were chosen in order to restrict the datasets to those genes that are most significantly affected by 20E and *EcRi*. The lower fold cutoff for the organ culture data reflects the observation that 20E responses tend to be reduced in organ culture when compared to the intact animal [17] (unpublished results). This is, most likely, due to the stress of dissection and *in vitro *culture. Microarray data from this study can be accessed at the National Center for Biotechnology Information Gene Expression Omnibus website [56], with accession numbers as follows: GSE3057 for the temporal array series; GSE3060 for the organ culture array series; and GSE3069 for the *EcRi *array series.

Comparisons were made between our microarray datasets and previously published microarray datasets using Microsoft Access. Each dataset was split into genes that are either upregulated or downregulated, represented by up or down arrows in Table 1. The *EcRi *datasets were constrained to at least a twofold change in expression level in order to reduce the 4,188 genes to more manageable numbers, resulting in 634 upregulated genes and 924 downregulated genes (Table 1). For the stress-regulated genes, only those genes that show at least a twofold change in expression with either paraquot, tunicamycin, or H_2_O_2 _treatment were used for comparison [43]. The 400 immune genes were split into 221 genes that showed consistent upregulation and 148 genes that showed consistent downregulation, eliminating 31 genes that showed an inconsistent profile under the conditions tested [44,47]. All other microarray datasets used for our comparison studies were as published (see Table 1 legend).

### Northern blot hybridizations

Total RNA was isolated using Trizol (Gibco) from staged animals or organ cultures, fractionated by formaldehyde gel electrophoresis, transferred to nylon membranes, and probed with radioactively labeled probes [27]. To facilitate comparisons, *w*^*1118 *^blots and *brat*^*k06028 *^blots where probed, washed, and exposed together. *rp49 *was used as a loading control. Probes were generated by PCR from genomic DNA using the following pairs of primers: *hairy *forward (F), 5'CAAATTGGAAAAGGCCGACA3'; *hairy *reverse (R), 5'AGAGAAACCCTAAGCGGCTT3'; *cabut *F, 5'CTCTTCTAGTAGCCAAGACG3'; *cabut *R, 5'GAGATTGGTTCTGATGCTGC3'; *sox box protein 14 *F, 5'TCAGCAAGGACGATAAGCAGC3'; *sox box protein 14 *R, 5'AGCTCCGTTGTTATCGTGTGC3'; *CG5249 *F, 5'ACGATGTGGATCCTGAGACG3'; *CG5249 *R, 5'GCTCCATCATCAGCATGTGC3'; *CG11275 *F, 5'ACACAGATTGCGTGTTCCACG3'; *CG11275 *R, 5'TGGACAACGTGACTCCATACG5'; *brain tumor *F, 5'TCTCCACGAACTGGAGAACG3'; *brain tumor *R, 5'TGATGGTGTGACTGTTGGTGG3'; *vrille *F, 5'ACAGTTGTTGGCATCGCTGC3'; *vrille *R, 5'GACAACAAGAAGGACGAGAGC3'.

## Additional data files

The following additional data are available with the online version of this paper. Additional data file 1 lists the 4,188 genes that change their expression ≥1.5-fold or ≤-1.5-fold in at least one time point in *EcRi *animals. Additional data file 2 lists 20E-regulated and 20E primary-response genes. Additional data file 3 is a figure showing Northern blot analysis of RNA samples isolated from *w*^*1118 *^or *EcRi *animals staged at -4, 0, or 4 hours relative to pupariation.

## Supplementary Material

Additional data file 1 The 4,188 genes that change their expression ≥1.5-fold in at least one time point in *EcRi *animals. Click here for file

Additional data file 2 20E-regulated and 20E primary-response genes. Click here for file

Additional data file 3 Northern blot analysis of RNA samples isolated from *w*^*1118 *^or *EcRi *animals staged at -4, 0, or 4 hours relative to pupariation. Click here for file

## Figures and Tables

**Table 1:** *EcRi *and 20E microarray gene sets compared to gene sets from published microarray studies

Microarrays		*EcRi*↑ (n = 634)	*EcRi*↓ (n = 924)	20E↑ (n = 411)	20E↓ (n = 68)
A	midgut;*EcR*↑ (n = 371)	19 (5.8E-01)	120 (8.4E-91)*	65 (1.3E-63)*	4 (9.7E-02)
B	midgut;*EcR*↓ (n = 292)	113 (1.2E-176)*	12 (8.2E-02)	3 (5.0E-02)	10 (3.1E-13)*
C	Steroid CD↑ (n = 916)	34 (2.1-01)	192 (5.3E-73)*	125 (1.7E-87)*	2 (2.3E-01)
D	Steroid CD↓ (n = 931)	53 (8.0E-02)	157 (9.3E-39)*	59 (2.3E-10)*	16 (2.3E-08)
E	Starv Sug↑ (n = 718)	120 (3.1E-58)*	87 (1.1E-09)	47 (4.5E-09)	12 (2.9E-06)
F	Starv Sug↓ (n = 667)	55 (4.3E-06)	90 (7.7E-13)*	32 (4.9E-03)	10 (1.5E-04)
G	Stress↑ (n = 253)	33 (5.5E-11)*	34 (1.1E-05)	12 (8.7E-02)	9 (1.4E-12)*
H	Stress↓ (n = 310)	87 (4.7E-90)*	25 (3.0E-01)	9 (9.7E-01)	3 (2.2E-01)
I	Immune↑ (n = 221)	56 (1.1E-50)*	29 (8.8E-05)	14 (2.6E-03)	13 (3.5E-31)*
J	Immune↓ (n = 148)	40 (7.5E-40)*	17 (1.7E-02)	4 (8.6E-01)	3 (6.8E-03)

This table depicts a comparison of *EcRi *(columns 1 and 2) and 20E-final (columns 3 and 4) microarray data with five other gene sets: rows A and B, *EcR*-dependent genes expressed in the larval midgut [20]; rows C and D, cell death genes expressed in the larval salivary gland [37]; rows E and F, genes that change expression in response to starvation or a sugar diet [45]; rows G and H, genes that change expression in response to either paraquot, tunicamycin, or H_2_O_2 _[43]; and rows I and J, genes that change expression in response to bacterial insult [44]. Each gene set is divided into upregulated or downregulated genes as represented by the arrows, with the number of genes in each dataset represented by '(n =)'. The first number in each cell represents the number of overlapping genes between the two datasets being compared. The numbers within the parentheses in each cell represent a *p* value based on the χ^2 ^test that accounts for the differences between the observed and expected numbers. Correlations discussed in the text are marked with an asterisk (all those showing a *p* value ≤ E-10).

**Table 2:** 20E-induced, *EcR*-dependent genes

Gene	20E	20E primary	*EcRi *-4 h	*EcRi *0 h	*EcRi *4 h	Function
Eip74A*	11.0	12.6	-4.9	-4.5	-2.0	Transcription factor
Drosomycin-2	8.1		-4.5	-7.3	-14.5	Defense response
CG14664	4.7		-16.3	-22.9		n/o
GST-E3	3.7	3.2		-1.9		Glutathione transferase
Eip78C*	3.6	3.3	-6.2	-6.9	-1.9	Nuclear receptor
CG10444	3.4		-4.1	-7.1	-2.1	Na^+^-dependent multivitamin transporter
Punch	3.0	2.4	-3.1	-8.1	-2.2	GTP cyclohydrolase I
CG5171	3.0	2.7	-1.7	-1.5	-3.5	Trehalose-phosphatase
CG2444	3.0		-2.8	-2.4	2.3	n/o
Cyp18a1*	2.9	8.3	-2.3		-1.7	Cytochrome P450
CG5391	2.7		-5.2		3.1	n/o
MESR3	2.6		-1.5	-3.0	-1.6	Ras signaling
CG3714	2.6	2.3		-1.7		Nicotinate phosphoribosyltransferase
rdgBbeta	2.5	4.6		-2.2	-2.4	Phosphatidylinositol transporter
CG8501	2.4			-1.9	-1.8	n/o
l(3)00305	2.4	1.9		-1.6		Serine/threonine kinase
CG11737	2.4		-2.3	-2.0		n/o
Cabut	2.3	5.9	-1.9			Transcription factor
CG17834	2.3	1.6		-2.1	-4.4	n/o
Sox box protein 14	2.3	2.9	-1.5	-1.7		Transcription factor
JIL-1	2.2				-3.0	Protein serine/threonine kinase
CG18212	2.2			-2.1	-1.8	n/o
CG13252	2.2	2.0	-2.1	-2.4	-2.7	n/o
raspberry	2.2	1.7	-1.9	-2.2	-1.8	IMP dehydrogenase
supernumerary limbs	2.2			-3.1	-2.6	Ubiquitin-protein ligase
CG16995	2.1	1.5			-2.8	Defense response
lamina ancestor	2.1	2.9	-1.6	-3.1	-2.4	n/o
CG33090	2.1			-3.9	-1.5	n/o
CG7701	2.1			-1.6		n/o
rhea	2.1			-1.9	-2.2	Actin binding
Ptp52F	2.1	2.2	-2.4	-1.6		Protein tyrosine phosphatase
CG4822	2.1	1.4		-3.1	-2.2	ATPase activity
big brain	2.1				-2.4	Water channel
brain tumor	2.0	2.9		-1.7		Translation regulator
CG11529	2.0		-7.1	-2.2	2.0	Serine-type endopeptidase
CG11509	2.0	1.9			-1.6	n/o
CG3348	2.0	4.5	-3.0	-3.1	-1.7	n/o
CG9989	2.0		-1.5	-2.2	-2.5	n/o
blue cheese	1.9	2.0		-2.0	-1.6	Intracellular protein transport
CG14073	1.9	2.4			-1.5	Ankyrin-like
kruppel homolog 1*	1.9	3.5		-1.8	-2.2	Transcription factor
CRMP	1.8	2.6		-2.3	-3.2	Dihydropyrimidinase
CG9801	1.8	3.1		-4.5	-5.7	n/o
CG8483	1.8	5.4	-3.0	-2.8		Defense response
CG9005	1.8	2.4	-1.8	-1.8	-1.8	Cell adhesion
vrille	1.6	3.5			-1.6	Transcription factor
CG1342	1.6	2.4		-2.6	-8.5	Proteinase inhibitor I4, serpin
DHR3*	1.6	2.3			-2.9	Nuclear receptor
Eip75B*	1.5	2.8	-1.6			Nuclear receptor
hairy		4.4		-1.8	-1.6	Transcription factor
CG9192		3.3			-1.7	n/o
CG5249		3.0			-1.5	Transcription factor
black		2.7	-3.0	-7.0		Glutamate decarboxylase 2
CG2016		2.1	-2.0	-2.0	-2.0	n/o
CG12539		2.1	-1.7		-1.6	Glucose dehydrogenase
CG8788		2.0	-1.5	-2.5	-1.8	n/o

Genes that show at least a twofold induction with either 20E alone (20E), or 20E + cycloheximide (20E primary) are listed in the order of their fold-induction by 20E alone. Downregulation of these genes upon *EcR *RNAi is shown for each time point, -4, 0, or 4 hours relative to puparium formation. Function is inferred from gene ontology on FlyBase. [40]. Asterisks denote previously identified 20E-regulated genes. n/o, no ontology.
