# Supplementary material for: The genomic response to 20-hydroxyecdysone at the onset of Drosophila metamorphosis
Source: Genome Biol. 2005 Nov 21;6(12):R99. doi: 10.1186/gb-2005-6-12-r99 (PMC1414087; doi:10.1186/gb-2005-6-12-r99)
Supplement: Additional data file 3 — Northern blot analysis of RNA samples isolated from w1118 or EcRi animals staged at -4, 0, or 4 hours relative to pupariation. [file gb-2005-6-12-r99-S3.doc]

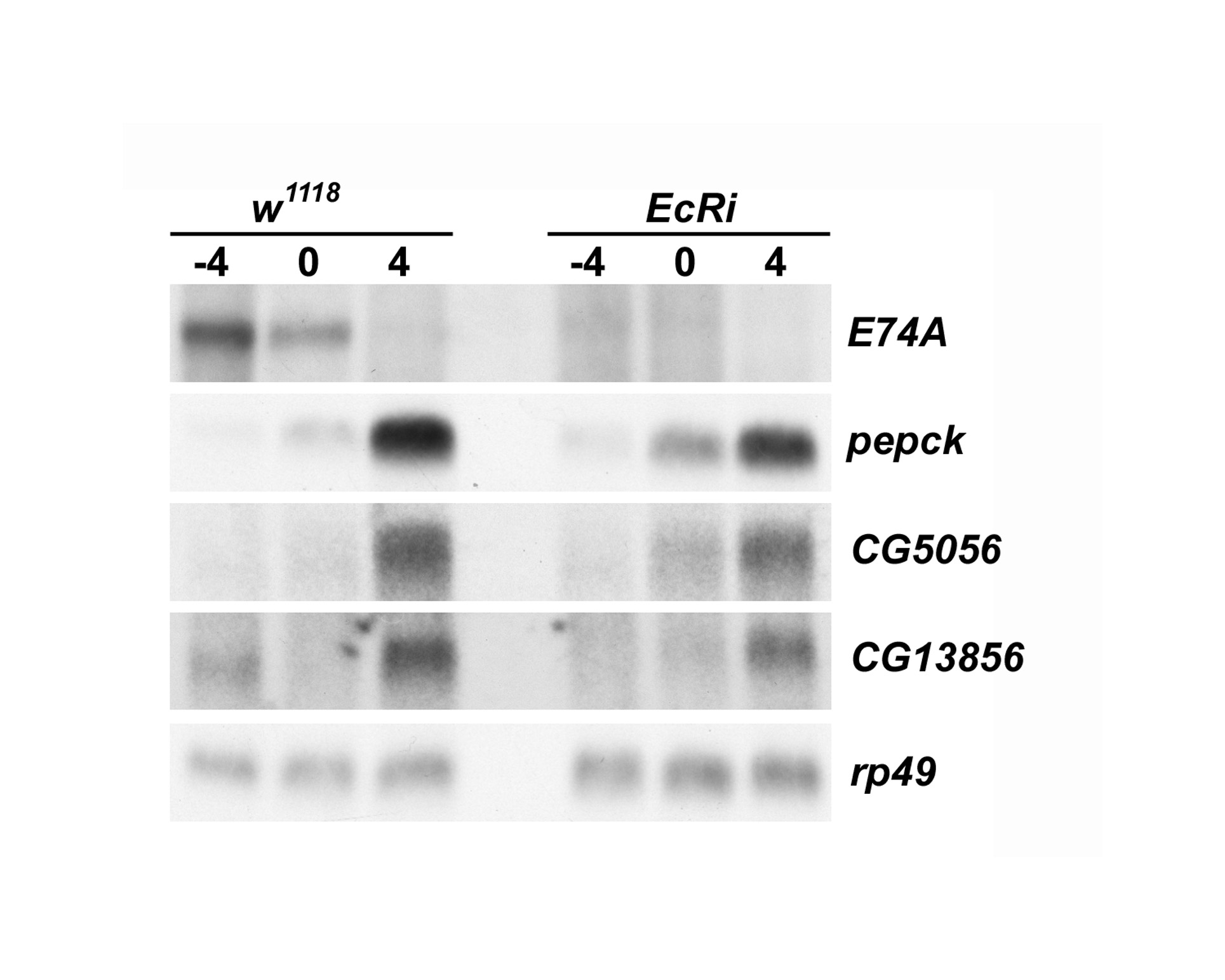


**Additional data 3:** Northern blot analysis of RNA samples isolated from *w^1118^* or *EcRi* animals staged at -4, 0, or 4 hours relative to pupariation. The *E74A* control is expressed in late third instar larvae (-4 hrs) and newly formed prepupae (0 hr), as expected, but not expressed in *EcRi* animals. In contrast, *pepck*, *CG5056*, and *CG13856* are induced normally at puparium formation in both *w^1118^* control and *EcRi* animals. Hybridization to detect *rp49* mRNA is included as a control for loading and transfer. The probe for *E74A* was generated by restriction digestion of a plasmid containing the *E74* cDNA (pBS-E74BcDNA) using BglII and SalI. The probe for *pepck* was isolated by restriction digestion of a plasmid containing the *pepck* cDNA (RH57538, Open Biosystems) using EcoRI and XhoI. Probes for CG5056 and CG13856 were generated by PCR from genomic DNA using the following pairs of primers: CG5065 L: 5’GAAAGCAACCTCCAGGACAA3’; R: 5’TACGTCCCGCATAAAACTGG3’; CG13856 L: 5’TGGCATCGCTAATCATCTTG3’; R: 5’CCGTCAGACCGGACTTTTTA3’
